# External Quality Assessment of Molecular Testing for HLA-B*58:01 Allele in Shanghai

**DOI:** 10.3390/diagnostics16071005

**Published:** 2026-03-27

**Authors:** Jing Quan, Pengyin Zhang, Yanqun Xiao, Xiaobo Hu, Yun Bao

**Affiliations:** Shanghai Center for Clinical Laboratory, 528 Hongshan Road, Shanghai 200126, China

**Keywords:** hyperuricemia, allopurinol, HLA-B*58:01 genotyping, external quality assessment (EQA)

## Abstract

**Background/Objectives:** Allopurinol, a first-line drug for gout and hyperuricemia, carries a risk of severe cutaneous adverse reactions (SCARs). Studies have established a strong association between HLA-B*58:01 and this adverse reaction. Although pre-treatment genotyping is recommended, the reliability of HLA-B*58:01 genetic testing varies across laboratories. This study aims to assess the performance of HLA-B*58:01 genetic testing of clinical laboratories in Shanghai through an External Quality Assessment (EQA) program, evaluating accuracy and standardization. **Methods**: The EQA program was carried out twice a year in 2023 and 2024. Each EQA sample panel consisted of five distinct samples, including HLA-B*58:01 allele-positive and -negative cell cultures. Sample panels were distributed to clinical laboratories through the cold chain system and results were analyzed and scored. **Results**: EQA samples used in this study were optimized for evaluating current HLA-B*58:01 genotyping assays, and the EQA samples were proved to be homogeneous and stable through each EQA period. In 2023, 17 and 16 clinical laboratories participated in the two EQA schemes; in 2024, 34 and 33 laboratories participated. A total of 14/17 (82.4%), 16/16 (100%), 33/34 (97.1%), and 33/33 (100%) laboratories achieved “optimal” scores. **Conclusions**: EQA results indicate that most of clinical laboratories in Shanghai exhibit constantly satisfactory performance for HLA-B*58:01 genotyping. However, a few laboratories still need further improvement. Additionally, EQA has demonstrated to be an important method for monitoring clinical laboratories’ performance.

## 1. Introduction

Gout is a prevalent form of inflammatory arthritis resulting from the deposition of monosodium urate crystals in joints and periarticular tissues [1]. This condition is pathophysiologically linked to chronic hyperuricemia, which serves as the primary metabolic precursor for gout development [2]. Globally, gout exhibits significant epidemiological variability across regions, with rising incidence rates attributed to lifestyle modifications and dietary changes. The 2017 Global Burden of Disease (GBD) study reported an increase in gout prevalence from 7.5‰ to 7.9‰ in men and 2.3‰ to 2.5‰ in women worldwide [3,4,5,6]. In China, data obtained from meta-analysis suggest that the prevalence of hyperuricemia is 13.3% and the prevalence of gout is around 1.1% [7,8].

The cornerstone of gout management involves effectively reducing serum uric acid levels to prevent its chronic deposition in renal tissues and musculoskeletal joints, thereby mitigating risks of renal impairment and secondary comorbidities [9]. Allopurinol, a xanthine oxidase inhibitor, suppresses the metabolic conversion of hypoxanthine and xanthine to uric acid, serving as a first-line therapeutic agent for gout by curtailing uric acid biosynthesis [10,11]. However, clinical application of allopurinol is associated with severe cutaneous adverse reactions (SCARs), especially allopurinol hypersensitivity syndrome (AHS), which including life-threatening conditions such as the Stevens–Johnson syndrome (SJS) and toxic epidermal necrolysis (TEN) [12].

Notably, pharmacogenomic studies have established a robust correlation between AHS susceptibility and the HLA-B*58:01 allele. Individuals with the HLA-B*58:01 allele exhibit an 80–97-fold increased risk of developing SJS/TEN compared to HLA-B*58:01-negative allopurinol users [13]. The allele frequency of HLA-B*58:01 demonstrates pronounced ethnic heterogeneity, with a particularly high prevalence in East Asian populations: Han Chinese (13.3–20.4%), Korean (12.2%), and Thai (8.1%). In contrast, lower frequencies are observed in Japanese (0.6%) and European populations (1.5–5.2%) [14]. These findings underscore the clinical imperative for HLA-B*58:01 genotyping prior to allopurinol initiation. Given this risk stratification, the American College of Rheumatology (ACR) and the Clinical Pharmacogenetics Implementation Consortium (CPIC) strongly recommend HLA-B*58:01 genotyping prior to allopurinol initiation in high-risk populations to prevent AHS [15,16].

Different methodologies, including Polymerase Chain Reaction (PCR) and sequencing, are employed for HLA-B*58:01 genotyping in China. Utilization of commercial PCR-based kits for HLA-B*58:01 genotyping also varies among laboratories. However, accuracy and reliability have not been determined. The accuracy of these tests is paramount, as inaccuracies can result in misdiagnosis, inappropriate treatment decisions, and ultimately, harm to patients.

This study aims to examine the performance of clinical laboratories in HLA-B*58:01 genotyping through the External Quality Assessment (EQA), employing cell cultures as samples which allow us to evaluate the entire processing procedure. We will review the assays employed in HLA-B*58:01 genotyping, analyze the challenges associated with laboratories, and provide potential strategies to enhance the quality of genetic testing services.

## 2. Material and Method

### 2.1. EQA Panel Preparation

The panel systematically includes both negative and positive samples with gradient concentrations. Negative samples are used to verify detection specificity. Positive samples with high, medium, and low concentrations are used to assess sensitivity of different assays. EQA samples procured from Genewell Biotechnology (Shenzhen, China) were derived from two human B-lymphoblastoid cell lines: GCHL006 (positive for HLA-B*58:01) and GCHL015 (negative for HLA-B*58:01). Each EQA panel comprised five samples with one or two negative samples and three to four positive samples with varied cell concentrations. All panels were maintained at −20 °C until distribution.

### 2.2. Sample Validation

GCHL006 and GCHL015 cell lines were validated using two methods. First, sequence-based typing (SBT), the gold standard for *HLA* genotyping, was performed by Dishuo Becken Biotechnology (Shanghai, China), with analytical reports provided by Genewell Biotechnology (Beijing, China). In addition, all cell lines underwent validation by qPCR assay. Genomic DNA was isolated using a QIAamp DNA Blood Mini Kit (QIAGEN GmbH, Hilden, Germany) and qPCRs were carried out with a commercial kit (Kuang Yuan Diagnostics, Suzhou, China) on a QuantStudio 5 Real-Time PCR System (Thermo Fisher Scientific Inc., Waltham, MA, USA). Results were considered valid when the internal control Ct value was ≤20. HLA-B*58:01 genotyping was determined by calculating ΔCt = Ct(HLA-B*58:01) − Ct(*GAPDH*): samples with ΔCt ≤ 4 were classified as positive, and those with ΔCt > 4 as negative. This dual validation approach confirmed the accuracy and reliability of the cell lines used for preparing the EQA panels.

### 2.3. Sample Homogeneity and Stability

Prior to the first EQA scheme of 2023, one batch of weakly positive samples and one of negative samples were selected for homogeneity validation, and 10 samples of each batch were randomly chosen. These samples were tested using qualitative PCR (Kuangyuan Diagnostics, Suzhou, China), following the manufactures’ instructions, and each sample was tested twice.

Stability was verified at two time points: before the annual EQA distribution and after simulated transport conditions. Each time, 12 samples (6 weak-positive and 6 negative) from the same lot were randomly selected and analyzed by qPCR. A *t*-test was used to compare the data from the two time points. *p* > 0.05 indicates that the samples remained stable under the tested storage and shipping conditions.

### 2.4. Submission of Results

We required that the laboratories complete testing using routine clinical protocols, and the results were returned via an online system within 7 days after receiving samples. Laboratories were requested to return the HLA-B*58:01 genotyping results (either “positive” or “negative”). Completion of a questionnaire at the time of reporting the results was also requested, including questions about the instruments used, methods of detection, and manufacturers of the reagents.

### 2.5. Evaluation of Results

The results were scored according to a qualitative binary “positive” or “negative” result. The EQA panel comprised five samples. A score of 20 points was awarded for each result that was in agreement with the expected value, whereas no points were given for false negative or false positive results. We used a standard with a score of 100 points classified as ‘optimal’, <100 points and ≥80 points regarded as ‘compliant’, and scores of  <80 points (>1 incorrect result) considered ‘non-compliant’. Laboratories that submitted error results were required to conduct a root cause analysis in accordance with the ISO 15189 standard [17]. This process follows a standardized workflow: describing the non-conformity, analyzing the underlying cause, planning corrective actions, implementing improvements, and verifying the effectiveness of the measures taken.

### 2.6. Statistical Analysis

In this study, the F-test (one-way analysis of variance, ANOVA) was employed to analyze the homogeneity of EQA samples. This method efficiently determines whether systematic differences exist across the entire batch by comparing inter-group variance with intra-group variance. If the calculated F-value is less than the critical F-value, the sample batch was considered homogeneous; otherwise, it was deemed non-homogeneous. The stability of EQA samples was assessed using a paired *t*-test, which evaluates temporal consistency by comparing mean measurement differences in the same batch at different time points. Stability was considered acceptable when CV% < 5% and *p* > 0.05.

## 3. Results

### 3.1. Validation of the Samples

**Figure 1:**
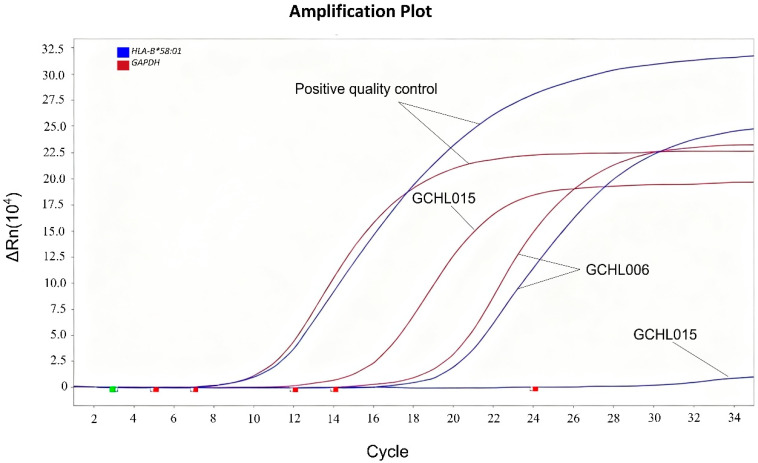
Validation of HLA-B*58:01 genotyping in cell lines by qPCR.

According to the SBT results, cell lines GCHL006 and GCHL015 were confirmed to be HLA-B*58:01-positive and HLA-B*58:01-negative, respectively (Table 1). The genotypes were further validated by qPCR (Figure 1). The results obtained from SBT and qPCR results were consistent for both cell lines.

Each EQA panel comprised five samples: 3–4 HLA-B*58:01-positive samples of varied cell concentrations, along with 1–2 HLA-B*58:01-negative samples (Table 2).

Homogeneity and stability testing by qPCR demonstrated 100% concordance between the observed and expected results for all samples, confirming the excellent homogeneity (Table S1) and simultaneous stability (Table S2) of the EQA samples.

In the amplification plot, the blue line represents the HLA-B*58:01 target gene, and the red line represents the *GAPDH* internal control gene. All samples had internal control Ct values ≤ 20, confirming effective DNA extraction and amplification. Genotyping was determined based on the ΔCt value (Ct(HLA-B*58:01)–Ct(*GAPDH*)). A sample was classified as positive if ΔCt ≤ 4, and as negative if ΔCt > 4. Based on this criterion, GCHL006 was identified as positive and GCHL015 as negative for the HLA-B*58:01 allele.

### 3.2. Participants and Assays

This study conducted four rounds of the EQA program, with 17, 16, 34, and 33 participating laboratories in each round, respectively. All laboratories submitted their results within the specified deadline of 7 days. qPCR was the most applied methodology, followed by sequencing and other assays such as MALDI-TOF-MS and Luminex xMAP.

Participating laboratories could be stratified into two categories: public hospital-affiliated (clinical laboratory or pharmacy department) and commercial (diagnostic center or laboratory). The number of participants in the public hospital category across the four rounds was 8, 8, 16, and 16, respectively, and in the commercial category, 9, 8, 18, and 17, respectively. The number and proportion of ISO 15189-accredited laboratories in each round were 6 (35.3%), 6 (37.5%), 19 (55.9%), and 19 (57.6%), respectively.

In the first round of the EQA program, four laboratories (4/17, 23.5%) had never previously participated in the HLA-B*58:01 genotyping EQA scheme organized by the Shanghai Center for Clinical Laboratory (SCCL). No new laboratories participated in the second or fourth rounds. In the third round, the number of laboratories participating in the SCCL EQA scheme for the first time increased to 15 (15/34, 44.1%).

### 3.3. Laboratory Performance

**Figure 2:**
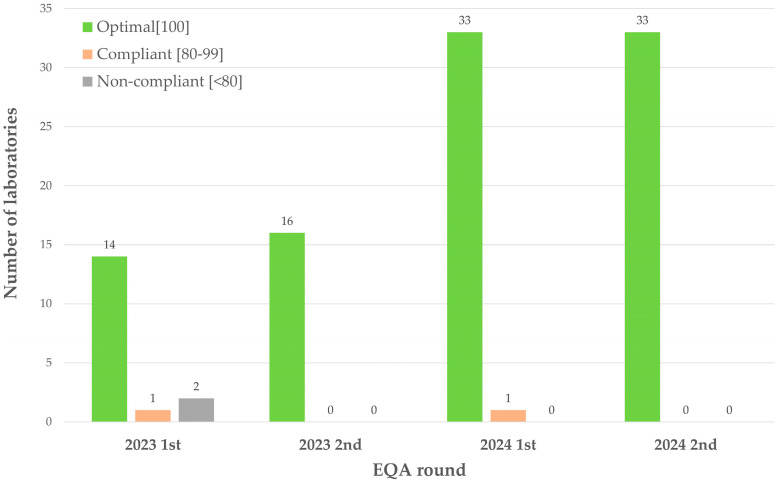
Distribution of laboratory performance across four EQA rounds.

In this study, although the participating clinical laboratories used different detection platforms (such as qPCR, Applied Biosystems by Thermo Fisher Scientific, Foster City, CA, USA; Illumina sequencers, Illumina, Inc., San Diego, CA, USA; Luminex 200, Luminex Corporation, Austin, TX, USA, etc.), the same set of evaluation criteria was applied to all detection methods. This is because HLA-B*58:01 gene typing is a “yes/no” qualitative determination—the result only needs to be categorized as positive or negative. In the four EQA rounds, the rates of laboratories achieving an “optimal” rating were 82.4%, 100%, 97.1%, and 100%, respectively (Figure 2). The Cochran–Armitage trend test showed a significant upward trend across rounds (*p* < 0.05), indicating that under a standardized assessment system, the testing capabilities of all laboratories were effectively enhanced through continuous participation in the EQA program, and the results across different platforms were comparable.

**Figure 3:**
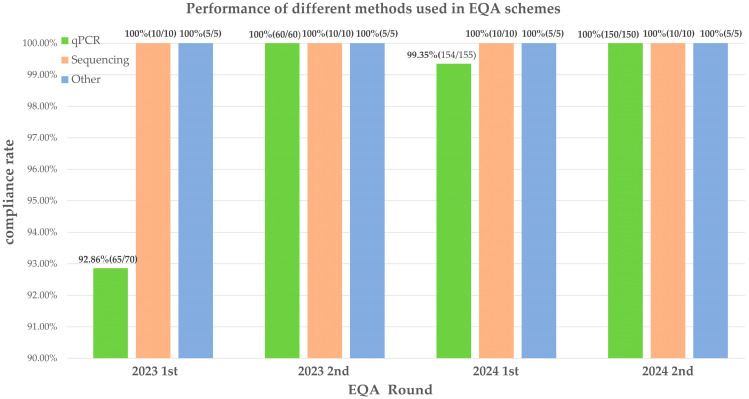
Performance of different methods used in EQA schemes.

qPCR emerged as the predominant detection methodology across four EQA cycles, demonstrating progressive improvement in concordance rates from 92.86% to 100%. Sequencing-based methods (sanger/next-generation sequencing) maintained 100% diagnostic accuracy throughout all assessments (Figure 3). Since error rates were higher in the first round, while few non-conformities occurred in subsequent rounds, we compared first-round error rates between the two platforms. qPCR tested 70 samples with 5 errors (7.14%) and sequencing tested 10 samples with no errors (0%). Fisher’s exact test showed no statistically significant difference (*p* > 0.05), indicating that the performance of the two platforms in this EQA round did not differ significantly.

This figure shows the number of laboratories in each rating category across different rounds. Green represents optimal (score 100), orange represents compliant (score 80–99), and red represents non-compliant (score < 80). The *x*-axis indicates the EQA round, and the *y*-axis represents the number of laboratories.

This figure shows the sample accuracy of qPCR, sequencing, and other testing platforms across four rounds of EQA. The *x*-axis represents the EQA round, and the *y*-axis represents the accuracy (%). Different colors represent different testing platforms: blue for qPCR, orange for sequencing, and green for other methods.

Each laboratory received 5 samples, the total number of samples analyzed in this EQA round is n × 5.

### 3.4. Distribution of Laboratory Types

High optimal performance rates were achieved by both ISO 15189-accredited and non-accredited laboratories across all four EQA rounds (Table 3). In the rounds with non-conformities (2023 1st and 2024 1st), Fisher’s exact test showed no significant difference between the groups (*p* > 0.05).

Analysis of first-time participating laboratories showed that their performance was strongly associated with prior EQA experience. In the first round, only 1 of 4 first-time participants (25%) achieved an optimal score, compared to all 13 laboratories with prior experience (100%). This difference was statistically significant (*p* < 0.05).

Notably, all laboratories that had non-conforming EQA results and implemented corrective actions following standardized procedures achieved 100% conformity in subsequent rounds. Furthermore, laboratories that had participated in the EQA scheme before 2023 maintained 100% conformity in every round. This demonstrates that regular participation in EQA programs helps laboratories improve and sustain testing quality.

## 4. Discussion

The human leukocyte antigen (HLA) system [18], encoded by the major histocompatibility complex (MHC) located on chromosome 6p21.3 [19], represents one of the most polymorphic genetic systems in humans, with over 35,000 documented alleles (IPD-IMGT/HLA). The HLA-B*58:01 allele, in particular, exhibits remarkable diversity, with 43 distinct subtypes identified through next-generation sequencing (NGS). This sequence heterogeneity demands specialized genotyping approaches that exceed the capabilities of conventional single-nucleotide polymorphism (SNP) detection.

Current methodologies for HLA-B*58:01 detection primarily include sequence-based typing (SBT), quantitative PCR (qPCR), as well as sequence-specific oligo nucleotide probing (SSOP). SSOP enables cost-efficient, intermediate-resolution HLA typing suitable for batch processing. But its clinical adoption is limited by high cross-reactivity and inadequate rare allele detection [20]. SBT, regarded as the gold standard for HLA genotyping, achieves 100% analytical accuracy by aligning sequencing results with the IPD-IMGT/HLA database. Despite its diagnostic precision, SBT is hampered by high operational costs and prolonged turnaround times [21,22], limiting its utility in high-volume clinical environments. In contrast, qPCR using TaqMan probes enables rapid, high-throughput detection at a lower costs [23], making it the preferred approach for routine clinical testing.

EQA serves as an essential component of the clinical laboratory governance framework, effectively enhancing and standardizing testing accuracy [24,25,26]. An EQA program was implemented to evaluate the performance of clinical laboratories in detecting the HLA-B*58:01 allele. EQA programs for this marker have been established in South Korea, the United Kingdom, and by China’s National Center for Clinical Laboratories (NCCL). Results from these programs indicate that laboratories generally achieve high levels of genotyping accuracy. However, differences in the types of EQA samples used across these programs limit the direct comparability of their findings. Specifically, South Korea and the UK utilize clinical blood samples, which suffer from poor uniformity and are prone to degradation [27,28]. In contrast, China’s NCCL uses genomic DNA, which, while stable, fails to monitor the DNA extraction step. This study employed a commercially available lymphoblastoid cell suspension as an EQA sample. This material combines both uniformity and stability, effectively mimics clinical blood samples, and enables comprehensive monitoring of the entire analytical workflow, from DNA extraction to PCR amplification. Four consecutive rounds of EQA have validated its consistent performance and clinical applicability, establishing it as an ideal material for HLA genotyping EQA.

This study analyzes four rounds of the HLA-B*58:01 EQA program conducted in Shanghai from 2023 to 2024, systematically evaluating the testing performance and trends of participating laboratories. The number of participants increased from 17 in the first round to 33 in the fourth round, reflecting the growing adoption of this test in clinical practice. More importantly, laboratory performance showed a significant and continuous upward trend across the four EQA rounds, demonstrating that sustained participation in a standardized EQA program effectively consolidates and enhances laboratory testing capabilities. We also compared test capability between different methods and found F-test results (*p* > 0.05) showed no statistically significant difference in error rates between qPCR and sequencing methods across all EQA rounds which indicates that, with proper optimization, qPCR can achieve accuracy comparable to sequencing in clinical practice. Furthermore, the results reflect that EQA could be a key driver for continuous improvement across platforms. In terms of laboratory characteristics, the rates of achieving “optimal” performance in the first EQA round were 83.3% for ISO 15189-accredited laboratories and 81.8% for non-accredited laboratories. The F-test showed no statistically significant difference (*p* > 0.05). While accredited laboratories showed a slightly higher rate, the lack of significance may be attributed to the limited sample size. Additionally, accreditation status alone does not fully capture the experience level of individual operators, particularly during first-time EQA participation.

In contrast, prior EQA experience was more strongly associated with achieving optimal performance. All laboratories with previous program participation before 2023 attained full marks in the first round, compared to only 25% of first-time participants. This underscores the dual role of the EQA as both a competency assessment tool and a valuable learning opportunity. Continuous participation enables laboratories to become familiar with procedures, optimize workflows, and strengthen quality control awareness, thereby contributing to sustained improvements in testing quality.

Across four rounds of the EQA, four participating laboratories reported six non-conformities, including four false negatives and two false positives. Based on the standardized investigation procedures initiated by each laboratory, these errors were primarily attributed to three key categories:

(1) Cross-Contamination Between Samples: In the first EQA round, one laboratory misclassified two negative samples (#2314 and #2315) as positive. Traceability analysis by the laboratory confirmed contamination by a positive sample due to operator error. To effectively mitigate cross-contamination risk, laboratories are advised to maintain a strict unidirectional workflow, proceeding sequentially from reagent preparation to sample processing, amplification, and product analysis, to utilize filtered pipette tips to prevent aerosol carryover, and to perform centrifugation prior to cap opening to avoid liquid splashing.

(2) Insufficient DNA Isolation: During the third round of the EQA, one laboratory failed to detect a low-concentration positive sample (#2414). Analysis indicated that this failure to detect likely stemmed from either insufficient efficiency of the DNA extraction kit or operator variability, resulting in isolated genomic DNA concentrations under the detection limit. This underscores the critical importance of optimizing nucleic acid extraction protocols. It is recommended to implement high-efficiency DNA extraction reagents, ensure the employment of competent personnel, and mandate the review of results falling within the equivocal (gray) zone to minimize the risk of missed detection.

(3) Defective Reagent Performance: During the first round of the EQA, two false negative results (#2311 and #2312) were reported by one laboratory, due to the EQA samples having relatively low concentrations (ranging from 1 × 10^5^ to 1 × 10^6^ copies/mL). Further investigation revealed that the laboratory used a detection kit not approved by the National Medical Products Administration (NMPA). Its technical limitations were the principal cause of the false negative readings, with the following contributing factors: inadequate sensitivity, resulting in limited detection capability for low-concentration samples; poor reagent stability, leading to inconsistent results and poor reproducibility; and a lack of systematic performance validation, including critical parameters such as the limit of detection, concordance rate, and precision.

To prevent such issues, it is strongly recommended that laboratories prioritize the use of NMPA-approved reagent kits. If unapproved kits must be employed, comprehensive performance validation must be conducted prior to implementation. Key validation metrics should include the limit of detection, concordance rate, precision, and other essential performance indicators to ensure analytical reliability.

Additional contributing factors include reporting errors from EQA system misuse and sample mix-ups due to operational errors. To address these issues, implementation of dual-review mechanisms for result reporting and comprehensive sample tracking via Laboratory Information Systems (LIS) is recommended to ensure analytical accuracy throughout the testing workflow.

Accurate reporting of HLA-B*58:01 genotyping is fundamental to personalized medicine. Continuous EQA implementation ensures testing reliability and helps reduce the risk of severe adverse drug reactions. Beyond its local impact, this study provides a scalable framework applicable to other pharmacogenomic markers, such as HLA-B*15:02 and *CYP2C19*. This establishes a universal model for standardized pharmacogenomics practice and supports the advancement of precision medicine into routine clinical care.

## Figures and Tables

**Table 1:** HLA genotyping results of GCHL006 and GCHL015 cell lines by SBT.

Cell Line	HLA-A	HLA-B	HLA-C	HLA-DRB1	HLA-DQB1
GCHL006	02:XX, 33:03	39:01, 58:01	03:02, 07:02	03:01, 12:02	02:01, 03:01
GCHL015	11:01, 68:01	15:02, 13:02	06:02, 08:01	07:01, 12:02	02:02, 03:01

Comprehensive high-resolution HLA typing, encompassing the HLA-A, -B, -C, -DRB1, and -DQB1 loci, was carried out, and the results show that GCHL006 was positive for HLA-B*58:01 and GCHL015 was negative.

**Table 2:** The EQA sample panel compositions and detection concordance rates of HLA-B*58:01.

EQA Round	Sample No.	Cell Line	Cell Concentration (Cells/mL)	HLA-B*58:01 Genotyping	Concordance Rates (%)
2023 1st	2311	GCHL006	1 × 10^6^	*+*	94.12
	2312	GCHL006	5 × 10^5^	*+*	88.24
	2313	GCHL006	1 × 10^5^	*+*	100.00
	2314	GCHL015	1 × 10^6^	*-*	94.12
	2315	GCHL015	1 × 10^6^	*-*	94.12
2023 2nd	2321	GCHL006	5 × 10^5^	*+*	100.00
	2322	GCHL015	1 × 10^6^	*-*	100.00
	2323	GCHL006	1 × 10^5^	*+*	100.00
	2324	GCHL006	1 × 10^6^	*+*	100.00
	2325	GCHL006	5 × 10^5^	*+*	100.00
2024 1st	2411	GCHL006	6 × 10^6^	*+*	100.00
	2412	GCHL006	3 × 10^6^	*+*	100.00
	2413	GCHL006	3 × 10^6^	*+*	100.00
	2414	GCHL006	1 × 10^6^	*+*	97.06
	2415	GCHL015	3 × 10^6^	*-*	100.00
2024 2nd	2421	GCHL015	3 × 10^6^	*-*	100.00
	2422	GCHL006	3 × 10^6^	*+*	100.00
	2423	GCHL006	3 × 10^6^	*+*	100.00
	2424	GCHL006	1 × 10^6^	*+*	100.00
	2425	GCHL006	6 × 10^6^	*+*	100.00

Composition of the EQA samples used for HLA-B*58:01 genotyping from 2023 to 2024. Each sample panel consisted of five samples derived from cell lines GCHL006 (HLA-B*58:01-positive) and GCHL015 (HLA-B*58:01-negative). Cell concentrations varied from 1 × 10^6^ to 6 × 10^6^ cells/mL to assess assay performance across different sample inputs. GCHL006 and GCHL015 were validated by SBT and qPCR prior to EQA distribution.

**Table 3:** Laboratory performance by ISO 15189 accreditation status across four EQA rounds.

EQA Round	ISO 15189 Accreditation Status	No. of Labs	No. of Optimal
2023 1st	Accredited	6	5 (83.3%)
	Non-accredited	11	9 (81.8%)
2023 2nd	Accredited	6	6 (100%)
	Non-accredited	10	8 (100%)
2024 1st	Accredited	19	19 (100%)
	Non-accredited	15	14 (93.3%)
2024 2nd	Accredited	19	19 (100%)
	Non-accredited	18	18 (100%)

## Data Availability

The data presented in this study are available on request from the corresponding author due to privacy restrictions.

## Supplementary Materials

The following supporting information can be downloaded at: https://www.mdpi.com/article/10.3390/diagnostics16071005/s1, Table S1. Homogeneity validation results of the EQA panel by duplicate qPCR testing; Table S2. Temporal stability validation results of EQA panel.
