# Supplementary material for: External Quality Assessment of Molecular Testing for HLA-B*58:01 Allele in Shanghai
Source: Diagnostics (Basel). 2026 Mar 27;16(7):1005. doi: 10.3390/diagnostics16071005 (PMC13073982; doi:10.3390/diagnostics16071005)
Supplement: Supplementary file 1 [file diagnostics-16-01005-s001.zip › diagnostics-4177550-supplementary.pdf]

Table S1. Homogeneity validation results of the EQA panel by duplicate qPCR testing.

Sample no.	**weak-positive（2313）**				**negative（2315）**			
	**Ct value (*HLA-B*58:01*)**		**Ct value (*GAPDH*)**		**Ct value (*HLA-B*58:01*)**		**Ct value (*GAPDH*)**	
	**(Rep1)**	**(Rep2)**	**(Rep1)**	**(Rep2)**	**(Rep1)**	**(Rep2)**	**(Rep1)**	**(Rep2)**
1	19.66	19.83	19.72	19.89	/	/	17.95	18.26
2	20.05	20.32	19.81	19.95	/	/	18.82	18.49
3	20.53	20.35	20.11	19.96	/	/	18.31	18.04
4	20.16	20.22	19.79	19.75	/	/	17.96	18.33
5	19.81	20.34	20.05	20.22	/	/	18.58	18.41
6	19.94	20.00	19.97	19.70	/	/	18.81	18.35
7	20.21	20.73	20.07	20.31	/	/	18.57	18.38
8	20.31	20.06	20.10	19.97	/	/	18.73	18.51
9	20.17	20.34	19.89	19.72	/	/	18.58	18.71
10	19.71	19.96	19.96	19.73	/	/	18.76	18.59
**Mean Ct**	20.05	20.21	19.95	19.92	/	/	18.51	18.41
**CV (%)**	1.38	1.29	0.70	1.10	/	/	1.80	1.00
**F**	2.63		2.78		/		2.64	
**F-crit**	3.02		3.02		/		3.02	

The homogeneity of the EQA panel was confirmed by qPCR analysis of 20 randomly selected samples (10 weak-positive and 10 negative). The Ct values for both the *HLA-B*58:01* and the *GAPDH* were highly consistent between duplicate tests, with all CV＜ 5% and no statistically significant differences (F＜F-crit). The results perfectly matched the expected genotypes, demonstrating excellent panel homogeneity and assay reproducibility

Table S2. Temporal stability validation results of EQA panel.

**Sample no.**	**Ct value (*HLA-B*58:01*)**		**Ct value (*GAPDH*)**		**Ct value (*HLA-B*58:01*)**		**Ct value (*GAPDH*)**		**Ct value (*HLA-B*58:01*)**		**Ct value (*GAPDH*)**		**Ct value (*HLA-B*58:01*)**		**Ct value (*GAPDH*)**	
	**(2313-A)**	**(2313-B)**	**(2313-A)**	**(2313-B)**	**(2314-A)**	**(2314-B)**	**(2314-A)**	**(2314-B)**	**(2414-A)**	**(2414-B)**	**(2414-A)**	**(2414-B)**	**(2415-A)**	**(2415-B)**	**(2415-A)**	**(2415-B)**
**1**	19.56	19.37	19.74	19.38	/	/	17.20	16.88	19.98	20.21	19.73	20.06	/	/	19.53	19.18
**2**	19.47	19.31	19.11	19.22	/	/	17.47	16.99	20.06	20.24	19.84	20.07	/	/	19.11	19.64
**3**	19.17	19.23	19.28	19.12	/	/	17.30	16.96	20.36	20.13	20.75	20.29	/	/	19.22	19.75
**4**	19.97	19.95	19.71	19.82	/	/	17.65	17.65	20.13	20.11	20.32	19.89	/	/	19.32	19.52
**5**	19.91	19.56	19.28	18.94	/	/	16.95	17.7	19.95	20.36	19.84	20.06	/	/	19.44	18.94
**6**	19.69	19.72	19.44	19.28	/	/	16.83	16.22	20.14	19.89	19.84	19.98	/	/	19.34	18.98
**Mean Ct**	19.63	19.52	19.43	19.29			17.23	17.07	20.10	20.16	20.05	20.06			19.33	19.34
**CV (%)**	1.51	2.09	1.31	1.55			1.79	3.22	0.73	0.78	1.99	0.66			0.78	1.80
**P-value**	0.13		0.18				0.53		0.64		0.97				0.96	

Stability of the EQA panel was assessed at two time points: before the first annual distribution and after simulated transport. Samples from lot 2313 (weak-positive) and 2314 (negative) were tested in 2023. Lots 2414 (weak-positive) and 2415 (negative) were analyzed in 2024. The initial state (A) and post-shipment simulation (B) were compared. All CV＜5%. No statistically significant differences were observed ($P > 0.05$). All parameters met the stability criteria. The EQA panel demonstrated consistent stability during storage and shipping
